# Dichloromethane Extract from *Amburana cearensis* (Allemão) A.C. Sm. Seeds and Its Coumarin Reduce ROS Production and Protect PC12 Cells Against Glutamate Excitotoxicity and Oxygen-Glucose Deprivation

**DOI:** 10.3390/antiox14040440

**Published:** 2025-04-05

**Authors:** Flávia Santos Sanches, Florisvaldo da Silva Ramos, Cinthia Cristina de O. S. Costa, Ravena Pereira do Nascimento, Bruno Solano de Freitas Souza, Maria de Fátima Dias Costa, Silvia Lima Costa, Paulo R. Ribeiro, Rafael Short Ferreira, Victor Diogenes Amaral da Silva

**Affiliations:** 1 Laboratory of Neurochemistry and Cell Biology, Department of Biochemistry and Biophysics, Institute of Health Sciences, Federal University of Bahia (UFBA), Salvador 40110-902, Bahia, Brazil; flaviasanches@ufba.br (F.S.S.); fdsramos@ufba.br (F.d.S.R.); fatima@ufba.br (M.d.F.D.C.); costasl@ufba.br (S.L.C.); 2 Gonçalo Moniz Institute, Oswaldo Cruz Foundation (FIOCRUZ), Salvador 40296-710, Bahia, Brazil; 3 D’Or Institute for Research and Education (IDOR), Salvador 41253-190, Bahia, Brazil; 4 Metabolomics Research Group, Department of Organic Chemistry, Chemistry Institute, Federal University of Bahia (UFBA), Salvador 40110-902, Bahia, Brazil; pauloribeiro@ufba.br

**Keywords:** neural cell death, *Amburana cearensis*, coumarin, ERK/MAPK pathway, neuroprotection

## Abstract

*Amburana cearensis* is a plant native to Brazil used in folk medicine for the treatment of several pathological conditions including stroke. Previous research indicates that a dichloromethane extract of *A. cearensis* seeds (EDAC), rich in coumarins, protects neural cells against oxygen and glucose deprivation (OGD) and glutamate-induced stress. However, further studies are needed to elucidate the role of coumarin, in the protective effect of EDAC. Glutamatergic excitotoxicity is an important cause of neuronal loss involved in the pathogenesis of Alzheimer’s disease, Huntington’s disease, Parkinson’s disease, and ischemic stroke. Therefore, this study aimed to investigate the protective effects of coumarin isolated from EDAC against glutamate excitotoxicity in regulating MAPK pathway proteins and reactive oxygen species (ROS) production on PC12 cells. Furthermore, we aimed to investigate the protective effects of coumarin against cell death induced by OGD. We characterized the isolated compound from EDAC as coumarin by ^1^H and ^13^C-NMR. Thus, PC12 cells were exposed to OGD or glutamate (20 mM) and/or treated with EDAC or coumarin (500 μg/mL) for 24 h. Subsequently, cell viability was assessed by propidium iodide staining or by MTT test. Furthermore, the expression of MAPK pathway proteins was investigated by Western blot analysis and the expression of cleaved caspase-3 by immunofluorescence. Furthermore, reactive oxygen species (ROS) production was assessed by 2′,7′-dichlorofluorescein diacetate and CellROX. We observed that EDAC and coumarin were able to protect PC12 cells against OGD conditions. Moreover, EDAC totally inhibited the glutamate toxicity in PC12 cells. Meanwhile, coumarin mitigated the glutamate toxicity. Both were able to downregulate the expression of ERK1/2 and phosphorylated ERK and inhibit caspase-3 activation. EDAC and coumarin also prevented the increase of ROS induced by treatment with H_2_O_2_ or glutamate. Our results evidenced that coumarin from *A. cearensis* is antioxidative and is an important cytoprotective compound in EDAC against glutamate excitotoxicity or OGD injury.

## 1. Introduction

The prospection of neuroprotective compounds has been a strategy for the development of new therapies for neurodegenerative diseases such as Parkinson’s Disease, Alzheimer’s Disease [1] and Amyotrophic Lateral Sclerosis [2], as well as for cerebral ischemia [3]. Cerebral ischemia is characterized as an acute vascular injury within the Central Nervous System (CNS) due to arterial occlusion, resulting in diminished blood flow [4]. During an ischemic stroke, the disruption of blood supply to the brain significantly impairs the delivery of oxygen and nutrients to neuronal cells, which leads to neurological deficits and subsequent cell death [5]. Eighty-seven percent (87%) of stroke cases are classified as ischemic. Collectively, various forms of stroke impact approximately 12 million individuals, making it the second leading cause of death worldwide and affecting potentially one in four people [6].

Following the injury caused by an ischemic stroke, various cell death mechanisms are triggered by glutamate excitotoxicity, including excessive calcium (Ca^2+^) influx, mitochondrial dysfunction, and oxidative stress [7,8]. Apoptosis can be promoted by increased Ca^2+^ influx due to glutamate excitotoxicity and lactic acidosis, activating proteins that degrade DNA and lead to cell death [9]. Molecular changes related to cell death in the CNS due to ischemic damage also involve the activation of the MAPK pathway, which includes a family of mitogen-activated protein kinases (Ras, Raf, MEK, and ERK1/2) [10].

The current treatment for ischemic stroke is based on thrombolysis or thrombectomy [11]. The search for natural compounds with neuroprotective action has been one of the new pharmacological approaches investigated in the scientific community [12,13,14,15,16]. Studies have shown the potential pharmacological benefits of compounds from *Amburana cearensis* A. C. Smith seeds for neuroinflammation [17] and ischemic stroke treatment [18,19]. Amburoside A from *A. cearensis* trunk barks was antioxidant and neuroprotective in rat primary mesencephalic cells culture treated with 6-hydroxydopamine [20].

Commonly known as ‘umburana’, ‘cumaru’ or ‘cerejeira’, the *Amburana cearensis* (Allemao) A.C. Smith is a plant native of Brazil’s northeastern Caatinga region, traditionally used in folk medicine, particularly by Indigenous peoples, and is known for its potential to alleviate symptoms of asthma, bronchitis, cough, headache, and used for stroke treatment [21]. Preclinical studies have demonstrated its pharmacological activity, including neuroprotective effects [22] (Table 1). Previous studies conducted by our research group demonstrated the neuroprotective effects of *Amburana cearensis* seed extracts. Among these extracts, the dichloromethane extract (EDAC), rich in coumarin, showed neuroprotective action in in vitro models of glutamate excitotoxicity and acute ischemia [18,19]. Other studies showed an anti-inflammatory and antioxidative effect of coumarin obtained from an *A. cearensis* plant cultivated for seven months in lipopolysaccharide-(LPS-) stimulated BV2 microglia cells [17] (Table 1). However, the role of coumarin in the neuroprotective effect of EDAC against glutamate excitotoxicity and oxygen-glucose deprivation (OGD) is still not clear.

Therefore, this study aims to investigate the neuroprotective effect of coumarin from *A. cearensis* seed and its role in the neuroprotective effect of EDAC against OGD and glutamate-induced cell damage in PC12 cells.

## 2. Materials and Methods

### 2.1. Preparation of Extracts of A. cearensis Seeds (EDAC)

Seeds of *Amburana cearensis* were purchased from a general store in the city of Feira de Santana, Bahia, Brazil, and checked for authenticity by comparison with seeds deposited in the Herbarium of the Biology Institute of the Federal University of Bahia, with the number 13734. All methods with *A. cearensis* seeds were carried out in accordance with the guidelines and standards of the University of Bahia, under registration in the National System for the Management of Genetic Heritage and Associated Traditional Knowledge (SisGen) under the number A73B242. After screening, the seeds were weighed, packaged in Kraft paper, and dehydrated for 48 h at 45 °C in a forced ventilation oven. The dried material was ground into powder using a blade mill and stored in an amber container at room temperature. Extraction followed the increasing polarity of solvents: hexane and dichloromethane. The seed powder was placed in a 5 L flask and three macerations were performed for each solvent independently. Solvent exchanges occurred three times every 48 h, with homogenization every 24 h. The macerate was protected from light at room temperature. After maceration, the material was filtered through hydrophilic cotton and then filter paper, concentrated in a rotary evaporator at 1.6 g and 40 °C ± 10 °C. The extract was then placed in a Petri dish in a fume hood for three days to dry completely, after which it was stored in light-protected bottles at 4 °C. During maceration with dichloromethane, the material showed two distinct phases: a yellow liquid supernatant and a whitish solid phase. The phases were separated using 80 g filter paper. To isolate coumarin, recrystallization was performed by subjecting the solid phase to −4 °C for 30 min, followed by filtration. Nuclear magnetic resonance (^1^H and ^13^C-NMR) identified the final whitish solid as coumarin.

### 2.2. PC12 Cell Culture

PC12 cell lines are derived from pheochromocytoma of the adrenal medulla of rats and exhibit characteristics similar to neuronal cells. These cells are frequently used in studies of neurodegenerative diseases to evaluate the neurotoxic or neuroprotective activity of substances by analyzing effects on cell survival and protein expression levels [28]. PC12 cells were obtained from the ATCC cell bank. They were cultured in DMEM medium (Gibco, SP, Brazil) supplemented with 1 μg/L glucose, 10% Fetal Bovine Serum (FBS), 5% Horse Serum (HS) (Cultilab, São Paulo, Brazil), 1% penicillin and 1% streptomycin (Cultilab, SP, Brazil) and maintained in 100 mm polystyrene plates (Kasvi, Campinas, Brazil). Cells were plated in 96-well plates (1 × 10^4^ cells/well) for cell viability assays, 24-well plates (6 × 10^4^ cells/well) for immunofluorescence staining or cell viability assays, and 6-well plates (2 × 10^5^ cells/well) for protein extraction or measurement of reactive oxygen species. The cells were kept in an incubator in a humidified atmosphere with 5% CO_2_ at 37 °C.

### 2.3. Glutamate Excitotoxicity and Treatment with EDAC and Isolated Coumarin

The L-glutamic acid solution (Sigma RES5063G, St. Louis, MO, USA) was prepared on the day of cell treatment. The appropriate amount for treatment was weighed, dissolved in DMEM medium with serum, forming a stock solution of 40 mM, and its pH was adjusted to 7.0. Before treatment, the medium containing 40 mM glutamate was diluted to 5 mM, 10 mM, and 20 mM and filtered. The EDAC and coumarin were diluted in DMSO to 1000 mg/mL stock solutions. For cell treatment, the stock solutions were diluted in DMEM to final concentrations. The concentrations of EDAC and coumarin were 5 μg/mL, 50 μg/mL, and 500 μg/mL. After 24 h of plating, the medium was replaced with a fresh medium containing EDAC or coumarin and/or glutamate. The cells were treated with EDAC and coumarin separately. As a control condition, the cells were treated with fresh medium with DMSO (0.05%). The PC12 cells were maintained for 24 h in DMEM with 10% FBS, 5% HS, and 0.05% DMSO containing EDAC or coumarin, and/or glutamate.

### 2.4. Oxygen–Glucose Deprivation (OGD) and Treatment with EDAC and Isolated Coumarin

Oxygen and glucose deprivation (OGD) was adapted from Butt et al. [29]. PC12 cell lines were cultured in 24-well plates (6 × 10^4^ cells/well). On the next day, the cell medium was removed and replaced with artificial cerebrospinal fluid (aCSF). This is composed of sodium chloride (NaCl) at a concentration of 130.21 mM, monosodium phosphate (NaH_2_PO_4_) (1.08 mM), HEPES (8.56 mM), potassium chloride (KCl) (3 mM), calcium chloride (CaCl_2_) (2.24 mM), magnesium chloride (MgCl_2_) (1 mM) and sucrose (2.76 mM) for cells under OGD conditions, along with glucose (9.99 mM) for cells under normal oxygen and glucose conditions (OGN). The aCSF was prepared on the day of treatment and its pH was adjusted to 7.3. Two different experiments were performed: one group of cells was treated concomitantly with EDAC or coumarin (500 μg/mL) during deprivation, and another group of cells only received the treatments (EDAC or coumarin) after deprivation. Both groups received treatment during reperfusion. EDAC and coumarin were diluted in aCSF-sucrose for OGD and aCSF-glucose for OGN. Subsequently, the cells were exposed for 1 h to normal oxygen and glucose conditions (OGN) or oxygen-glucose deprivation (OGD). Cells in normoxia were maintained in an incubator at 37 °C with 5% CO_2_. Cells in OGD were placed in a hypoxia chamber containing 95% nitrogen (N_2_) and 5% CO_2_, and were kept in an incubator at 37 °C. After 1 h, the aCSF was removed and the cells in OGN and OGD were incubated in a DMEM medium with 10% FBS and 5% HS containing EDAC or coumarin at the same concentrations (500 μg/mL). After 24 h (reperfusion), the cell viability was analyzed using propidium iodide (PI).

### 2.5. Cell Viability by Propidium Iodide (PI)

For the viability analysis, after the 24 h period, the cells were exposed to propidium iodide (PI) (5 μL/mL) for 30 min in an incubator at 37 °C with 5% CO_2_. After this time, the cells were photographed using an Eclipse TS100 Inverted Fluorescence Microscope (Nikon Instruments Inc., Americas). PI can absorb light at a wavelength of 536 nm and emit at 617 nm. Five images of each well were captured for analysis using ImageJ 1.33u software (Wayne Rasband, National Institutes of Health, USA). In this software, the count of PI-positive cells, which exhibited red fluorescence, was performed. The experiment was repeated three times (n = 3). The percentage of dead cells was then calculated, and the average was plotted in the graphic.

### 2.6. Cell Viability by MTT

The cell viability was also evaluated in PC12 cells treated with glutamate and/or EDAC or coumarin in 96-well plates (1 × 10^4^ cells/well) using 3-(4,5-dimethylthiazol-2-yl)-2,5-diphenyltetrazolium bromide (MTT; Sigma, St. Louis, MO, USA). The MTT test is based on the principle of converting a yellow substrate of formazan to violet crystals by the mitochondrial dehydrogenases of living cells [30]. The MTT solution was diluted in DMEM medium and added to each well at a final concentration of 1 μg/mL. The plates were incubated for 2 h in a humidified atmosphere with 5% CO_2_ at 37 °C. Subsequently, to complete the dissolution of formazan crystals, the cells were lysed with 100 μL/well of lysis buffer containing 20% sodium dodecyl sulfate (SDS) and 50% dimethylformamide (DMF) at pH 4.7. After 12 h at room temperature, the optical absorbance of each sample was measured using a spectrophotometer at 595 nm. The experiment was repeated six times (n = 6).

### 2.7. Immunofluorescence

Cells were plated on 24-well plates (6 × 10^4^ cells/well) on sterile coverslips pre-coated with Poly-D-lysine for 30 min in a humidified atmosphere with 5% CO_2_ at 37 °C. The day after plating, the cells were treated with glutamate (20 mM), EDAC, and coumarin (500 μg/mL). After the treatment, the PC12 cells were washed three times with phosphate-buffered saline (PBS) and fixed with cold methanol for 10 min at 20 °C. After fixation, the cells were washed three times with PBS and permeabilized with 0.1% Triton diluted in PBS for 10 min at room temperature, followed by three washes with PBS. A blocking step was then performed using a solution containing 10% goat serum, 3% bovine serum albumin (BSA), and 0.01% Triton diluted in PBS for 1 h at room temperature. After blocking, immunostaining was carried out with a primary antibody against cleaved caspase-3 rabbit polyclonal antibody (1:10, Merck, AB3623, Burlington, MA, USA) diluted in the blocking solution and incubated overnight. The next day, the primary antibody was removed and the cells were washed three times with PBS for 5 min with slow agitation. Subsequently, a secondary antibody, Alexa Fluor 594-conjugated goat anti-rabbit (1:500, Thermo Fisher Scientific, A11012, Waltham, MA, USA), diluted in blocking solution, was applied and incubated for 2 h in the dark at room temperature. After incubation, the cells were washed three times with PBS, and nuclear staining was performed using DAPI (4′,6-diamidino-2-phenylindole) (100 μL/well) for 5 min. The cells were then washed again with PBS. The coverslips were mounted on slides with N-propyl-gallate 5% mounting medium (5 μL). The images were captured using a fluorescence microscope and digital camera (Leica DMIL Led Fluo Microscope and Leica DFC7000 T Camera with Leica Application Suite LAS X 5.3.0 software and LAS Overlay module for fluorescence). Relative fluorescence of caspase 3 in red was analyzed in the images using Fiji ImageJ software (Wayne Rasband, National Institutes of Health, USA). Three independent experiments were performed.

### 2.8. Western Blot

Cells were plated in 6-well plates (2 × 10^5^ cells/well) and treated with glutamate (20 mM), EDAC, and coumarin (500 μg/mL) for 24 h. The cells were then washed three times with PBS and lysed in extraction buffer containing 200 mg of SDS, 7.6 mg of EGTA, 2.4 g of urea, 50 μL of Triton X-100, 1250 μL of Tris-HCl buffer (pH 6.8), 100 μL of NP40, and protease inhibitors (Sigma P8340) at a concentration of 1 μL/mL. Protein quantification was adapted from Lowry et al. using the DC Protein Assay Kit (Bio-Rad, Hercules, CA, USA) [31]. For electrophoresis, a 10% polyacrylamide stacking gel was used. For analysis of ERK1/2 and p-ERK, 10 μg was loaded per well. Electrophoresis was performed at 80 V for 30 min, followed by 100 V for 2 h. The proteins were then transferred to a polyvinylidene fluoride (PVDF, Immobilon-P, Millipore) membrane at 100 V for 1 h. The protein loading was confirmed by staining the membranes with Ponceau S red (Sigma P3504). Subsequently, the membranes were blocked for 1 h at room temperature in Tris-buffered saline (TBS) containing 20 mmol/L Tris (pH 7.5), 0.05% Tween 20 (TBS-T), and 5% skimmed milk powder. The membranes were then incubated with primary antibodies: anti-ERK1/2 rabbit antibody (1:1000, Santa Cruz, SC93) and anti-phospho-p44/42-MAPK-ERK1/2-Thr202/Tyr204 rabbit antibody (1:1000, Cell Signaling Technology, #9101) in blocking solution overnight. The next day, the membranes were washed with TBS-T and incubated with secondary IgG anti-rabbit antibody conjugated with horseradish peroxidase (HRP) (1:1000). For loading control, the primary anti-alpha-tubulin mouse antibody (1:1000, Santa Cruz, SC23948) was used, along with IgG anti-mouse antibody conjugated with horseradish peroxidase as the secondary antibody. For membrane detection, the immunoreactive bands were visualized by chemiluminescence using the Immune Start HRP Substrate Kit (Bio-Rad) on the ImageQuant LAS 500 (GE 29005063). Quantification was obtained by densitometric scanning (ScanJet 4C, Hewlett Packard) of three independent experiments and analyzed using ImageJ 1.33u software (Wayne Rasband, National Institutes of Health, USA).

### 2.9. Quantitative Assay of Reactive Oxygen Species

PC12 cells were plated in 6-well plates (2 × 10^5^ cells/well) and treated concurrently with 10 μM hydrogen peroxide (H_2_O_2_) and/or EDAC (500 μg/mL) or CUM (500 μg/mL) for 1 h. After treatment, the cells were trypsinized, centrifuged for 5 min at 2000 rpm, and resuspended in 1 mL of PBS. The suspension was placed in black 96-well plates containing 195 μL of cells and 5 μL of 2′,7′-dichlorofluorescein diacetate (D6883, Sigma-Aldrich) at a concentration of 10 μM. The plate was kept in an incubator for 30 min, and fluorescence was evaluated using a spectrophotometer with an excitation wavelength of 502 nm and an emission wavelength of 523 nm. The experiment was repeated three times (n = 3).

The measurement of ROS levels was also analyzed after exposure to glutamate using CellROX™. PC12 cells were plated in 96-well plates at a density of 1 × 10^4^ cells/well and treated concurrently with 20 mM of glutamate and/or EDAC (500 μg/mL) or CUM (500 μg/mL). After 24 h of incubation, the cells were exposed to CellROX™ Green reagent at a final concentration of 5 μM for 1 h. The fluorescence was read using a Spectrophotometer (Varioskan™ LUX multimode microplate reader) with an excitation wavelength of 485 nm and an emission wavelength of 520 nm. The experiment was repeated three times (n = 3).

### 2.10. Statistical Analysis

The results were analyzed using the statistical program Graph Pad Prism 8.0.1 (CA, USA) based on the means of each group ± SD (standard deviation). The Kolmogorov-Smirnov normality test was performed, and all results were defined as parametric. Subsequently, One-way ANOVA followed by Tukey’s test for multiple comparisons was conducted. *p*-values ≤ 0.05 were considered as statistically significant.

## 3. Results

### 3.1. Characterization of Coumarin

**Figure 1:**
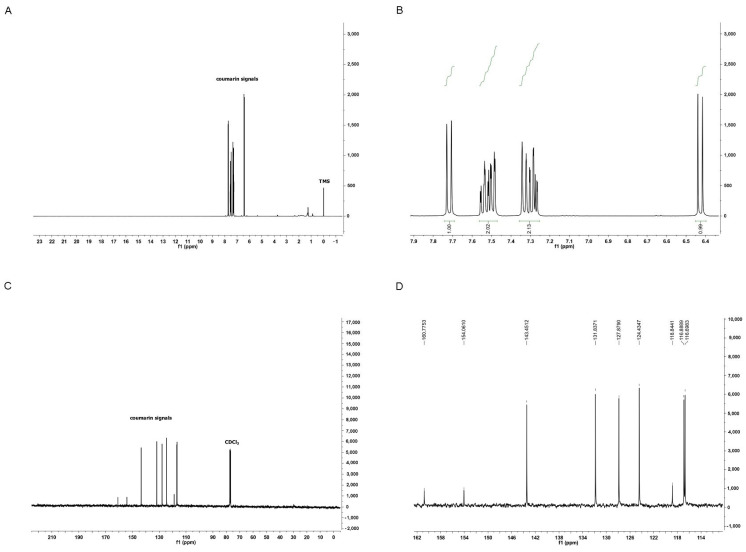
Chemical Characterization of Isolated Coumarin (2H-chromen-2-one). In (**A**), full ^1^H NMR spectrum of coumarin confirms its high purity. In (**B**), expansion of the ^1^H NMR spectrum of coumarin from 7.9 to 6.4 ppm. In (**C**), full ^13^C NMR spectrum of coumarin. In (**D**), expansion of the ^13^C NMR spectrum of coumarin from 162 to 114 ppm.

Coumarin was identified using proton nuclear magnetic resonance (^1^H NMR) and carbon-13 nuclear magnetic resonance (^13^C NMR) (Figure 1). This compound exhibited two doublets at 6.43 ppm (d, 1H, 9.56 Hz) and 7.72 ppm (d, 1H, 9.48 Hz), attributed to C2 and C3, respectively. It also displayed two doublets of doublets at 7.49 ppm (dd, 1H, 1.48 and 7.72 Hz) and 7.30 ppm (dd, 1H, 1.12 and 7.64 Hz), assigned to C5 and C6, respectively. Furthermore, it showed a triplet of doublets at 7.54 ppm (td, 1H, 1.2 and 8.68 Hz) attributed to C7, and a doublet of triplets at 7.34 ppm (dt, 1H, 0.44 and 8.36 Hz) assigned to C8. The NMR signals were consistent with the literature [32] and the 13C NMR spectrum of coumarin.

### 3.2. EDAC and Coumarin Protect PC12 Cells Against Oxygen and Glucose Deprivation (OGD)

**Figure 2:**
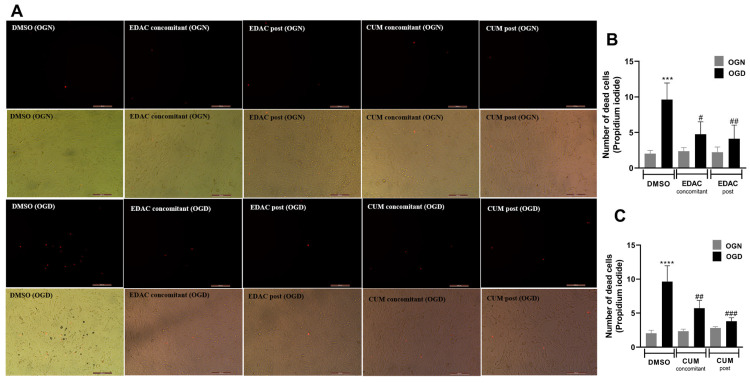
EDAC and coumarin protect PC12 cells exposed to oxygen and glucose deprivation (OGD) by propidium iodide (PI) staining. In (**A**), representative micrographs of cultures in different conditions. Cells in the control group received DMSO, while treated groups received EDAC (500 μg/mL) or coumarin (500 μg/mL) during deprivation (concomitant) or after 1 h OGD (post-OGD). Fluorescence microscopy images show the staining of dead cells by red fluorescence emission for the different treated groups. Magnification: 10×. In (**B**,**C**), gray graph bars represent the number of cells under normal oxygen and glucose conditions (OGN), and in black are cells under oxygen and glucose deprivation conditions (OGD). In (**B**), analysis of the number of dead cells treated with EDAC (500 μg/mL). Data represent Mean ± SD. In (**C**), analysis of the number of dead cells treated with coumarin (500 μg/mL). Data represent Mean ± SD. The experiment was repeated three times (n = 3). Data were tested for significance using one-way ANOVA; In (**B**) *** *p* ≤ 0.0003 and in (**C**) **** *p* ≤ 0.0001 indicate comparisons with the control OGN group, and # *p* ≤ 0.01, ## *p* ≤ 0.04 and ### *p* ≤ 0.0002 indicate comparisons with the control OGD group.

PC12 cells were exposed to oxygen and glucose deprivation (OGD) conditions or normal oxygen and glucose conditions (ONG) for 1 h in artificial cerebrospinal fluid (a-CSF). The cells were also treated with EDAC or coumarin (500 μg/mL) during deprivation (concomitant) and another group of cells only received the treatments after deprivation (post-OGD). After 1 h of OGD, the a-CSF was replaced with culture media containing treatments for 24 h. Following the treatment, cell viability was assessed via propidium iodide (PI) incorporation. It was observed that after 24 h of reperfusion, cell cultures in the OGD group exhibited higher mortality (9.6 ± 2.3), when compared to cell cultures in the ONG group (2 ± 0.45) (Figure 2).

After 24 h of reperfusion, it was found that the cell cultures treated with EDAC during the OGD (4.7 ± 1.7) showed a reduction in the number of dead cells when compared to the OGD group (9.6 ± 2.3). The reduction in the number of dead cells was also observed in the cultures treated with EDAC after the oxygen-glucose deprivation (4.1 ± 1.9) (Figure 2A,B).

Treatment with coumarin (500 μg/mL) demonstrated a reduction in cell death during both concomitant treatment and post-OGD treatment (Figure 2A,C). The concomitant treatment resulted in a cell death reduction of 5.7 ± 1.1 when compared to the OGD control (9.6 ± 2.3). The observed reduction was even more pronounced in the post-OGD treatment with coumarin, decreasing to (3.8 ± 0.51). Both concomitant and post-OGD coumarin treatments exhibited a difference when compared to the OGD control and no difference when compared to OGN (Figure 2A,C). Thus, it was concluded that coumarin was effective in protecting PC12 cells against damage induced by oxygen and glucose deprivation.

### 3.3. EDAC and Coumarin Protect PC12 Cells Against Glutamate Excitotoxicity

**Figure 3:**
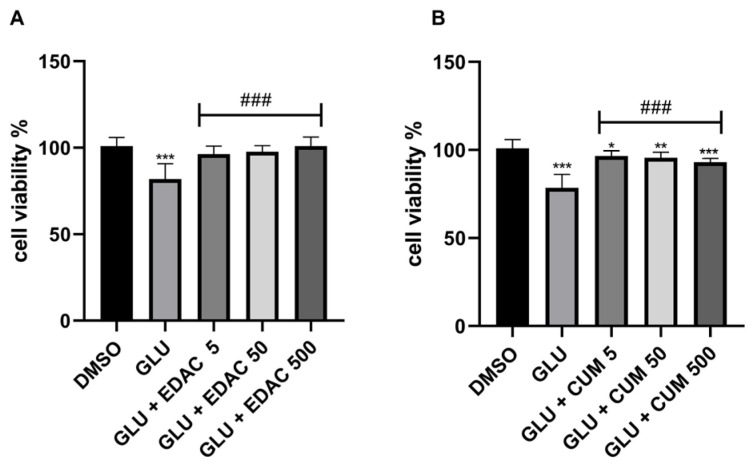
EDAC protects; meanwhile, coumarin mitigates PC12 cell death induced by glutamate excitotoxicity. In (**A**), MTT test in PC12 cells treated with glutamate (20 mM) and/or EDAC (5 µg/mL, 50 µg/mL, or 500 µg/mL) for 24 h. In (**B**), the MTT test in PC12 cells treated with glutamate (20 mM) and/or coumarin (5 µg/mL, 50 µg/mL, or 500 µg/mL) for 24 h. Data are expressed as a % of control and plotted as mean ± SD (n = 6 per experimental group). Data were tested for significance using one-way ANOVA, followed by Tukey’s post-hoc tests; * *p* ≤ 0.05, ** *p* ≤ 0.01, and *** *p* ≤ 0.001 indicate comparisons with the control group (DMSO), and ### *p* ≤ 0.001 indicate comparisons with 20 mM glutamate group.

After 24 h of concomitant treatment with glutamate (20 mM) and EDAC (5 μg/mL, 50 μg/mL, or 500 μg/mL), or coumarin (5 μg/mL, 50 μg/mL, or 500 μg/mL), cell viability was assessed using the MTT assay (Figure 3A,B). It was observed that treatment with 20 mM glutamate for 24 h induced a reduction in cell viability to 82 ± 9%, when compared with the control group (100 ± 5%). We also observed that all concentrations tested for EDAC were effective in protecting PC12 cells against glutamate-induced damage. The cell culture treated with glutamate and EDAC in the concentration of 5 μg/mL showed a cell viability of 96 ± 5%, while the cell culture treated with glutamate and 50 μg/mL EDAC exhibited a cell viability of 97 ± 4%. The cell culture treated with glutamate and 500 μg/mL EDAC presented cell viability of 101 ± 5%. Thus, it was concluded that all concentrations of EDAC were able to protect PC12 cells against glutamate-induced damage. Due to its superior results, the concentration of 500 μg/mL of EDAC was selected to treat cells in further analysis.

The cell culture treated with coumarin in all tested concentrations mitigated the reduction in cell viability induced by glutamate. The cell viability in culture treated with glutamate was 78.5 ± 8%; meanwhile, we observed that coumarins mitigated the cell death induced by glutamate in concomitant treatments. The cell viability in cultures treated with glutamate and coumarins in concentrations of 5 μg/mL, 50 μg/mL, and 500 μg/mL were 96.5 ± 3%, 95.62 ± 3%, and 93.0 ± 2%, respectively. However, these cell viability data were lower than that observed in control conditions (100 ± 5%).

### 3.4. EDAC and Coumarin Prevent Cysteinyl Aspartate Specific Proteinase-3 (Caspase-3) Activation in PC12 Cells Induced by Glutamate Excitotoxicity

**Figure 4:**
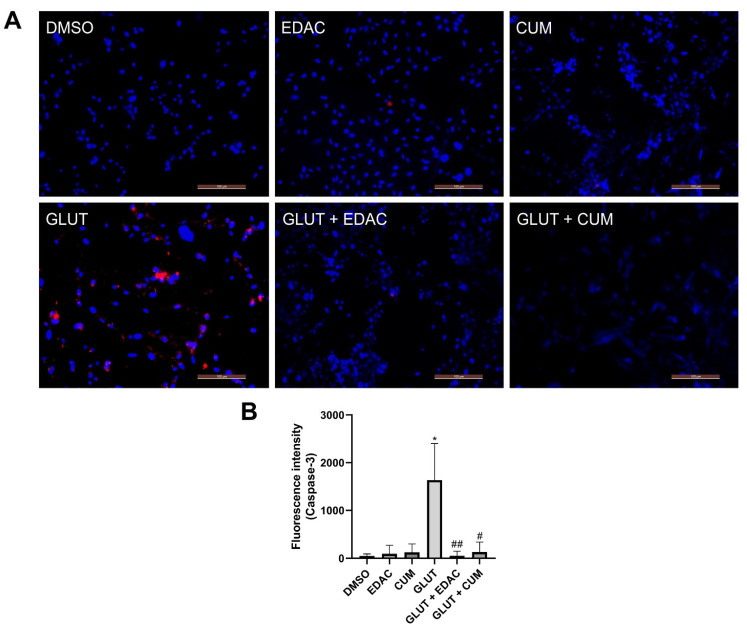
EDAC and coumarin prevent an increase in the cleaved caspase-3 expression in PC12 cells induced by glutamate. PC12 cells were treated with glutamate (20 mM) and/or EDAC or coumarin (500 µg/mL) for 24 h. In (**A**), a representation of caspase-3 expression (red) and cell nuclei stained with DAPI (blue). Magnification: 20x. In (**B**), the measurement of caspase-3 relative fluorescence in the groups analyzed. Data are expressed as mean ± SD (n = 3 per experimental group). Data were tested for significance using one-way ANOVA, followed by Tukey’s post-hoc tests; * *p* ≤ 0.01 indicates comparisons with the control group (DMSO), and # *p* ≤ 0.05 and ## *p* ≤ 0.01 indicate comparisons with 20 mM glutamate group.

After 24 h of treatment, the expression of cleaved caspase-3 in PC12 cells was analyzed using immunofluorescence (Figure 4). The results indicated an increase in cleaved caspase-3 relative fluorescence (1.635 ± 766) in the cell cultures treated with 20 mM glutamate when compared to the control group (48 ± 40). The groups treated with EDAC (91 ± 177) or coumarin (122 ± 175) (both 500 µg/mL) did not change the expression of cleaved caspase-3. The cell cultures treated concomitantly with EDAC (51 ± 91) or coumarin (125 ± 212) plus glutamate did not change the cleaved caspase-3 relative fluorescence when compared with the control group, which was different from the observed in the glutamate group.

### 3.5. EDAC and Coumarin Reduced the Expression of ERK and p-ERK in PC12 Cells

**Figure 5:**
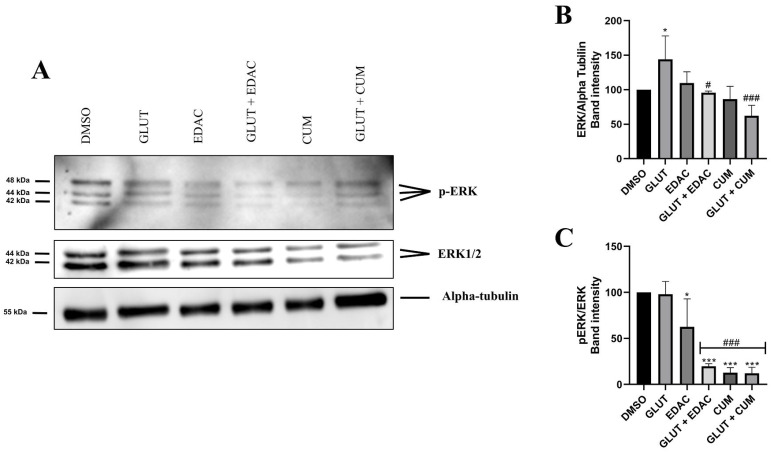
EDAC and coumarin reduced the expression of ERK1/2 and p-ERK in PC12 cells. PC12 cells were treated with glutamate (20 mM) and/or EDAC or coumarin (500 µg/mL) for 24 h. In (**A**), Western blot analysis shows bands immunoreactive for ERK1/2 (42/44KDa), *p*-ERK (42/44KDa), and alpha-tubulin (55KDa). In (**B**), the relative expression of ERK per Alpha-tubulin. In (**C**), relative expression of pERK per ERK. Data are expressed as mean ± SD (n = 3 per experimental group). Data were tested for significance using one-way ANOVA, followed by Tukey’s post-hoc tests; in (**B**,**C**), * *p* ≤ 0.05 and *** *p* ≤ 0.001 indicate comparisons with the control group (DMSO), # *p* ≤ 0.05 and ### *p* ≤ 0.001 indicate comparisons with 20 mM glutamate group.

Western blotting data demonstrated that 24 h of treatment with glutamate (20 mM) increased the expression of ERK (144 ± 33%) in PC12 cells when compared to the control conditions (100% ± 0) (Figure 5A,B). The increase in ERK promoted by glutamate was prevented by concomitant treatment with EDAC (96± 2.6%) or coumarin (62 ± 15%) (Figure 5A,B). Additionally, we observed that treatment with EDAC decreased the p-ERK expression in the absence (62.5 ± 30%) or in the presence of glutamate (19.7 ± 2.7%). A similar reduction in p-ERK expression was observed in the treatment with coumarin in the absence (12.7 ± 5%) or the presence of glutamate (12 ± 7%) (Figure 5A,C).

### 3.6. EDAC and Coumarin Decrease the Production of Reactive Oxygen Species (ROS) in PC12 Cells Treated with Hydrogen Peroxide (H_2_O_2_) or Glutamate

**Figure 6:**
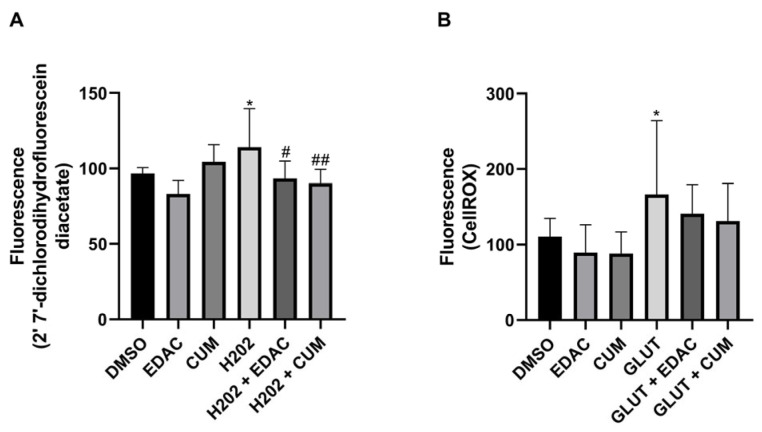
EDAC and coumarin reduce the production of reactive oxygen species (ROS) in PC12 cells exposed to hydrogen peroxide (H_2_O_2_) and glutamate. PC12 cells were treated with glutamate (20 mM) and/or EDAC or coumarin (500 µg/mL) for 24 h. In (**A**), Analysis of the production of reactive oxygen species (ROS) in PC12 cells using 2′ 7′-dichlorodihydrofluorescein diacetate. In (**B**), Analysis of ROS production in PC12 cells using CellROX. Data are expressed as mean ± SD (n = 3 per experimental group). Data were tested for significance using one-way ANOVA, followed by Tukey’s post-hoc tests; * *p* ≤ 0.05 indicates comparisons with the control group (DMSO), and # *p* ≤ 0.05 and ## *p* ≤ 0.01 indicate comparisons with H_2_O_2_ group or glutamate group.

To analyze the antioxidant effect of EDAC and coumarin against oxidative stress, the PC12 cells were exposed to H_2_O_2_ or glutamate. It was observed that 1 h of treatment with H_2_O_2_ (10 μM) increased the production of reactive oxygen species (ROS) (114 ± 26%) in PC12 cells when compared to the control condition (97 ± 4%), which was inhibited by concomitant treatment with EDAC (93 ± 11%) or coumarin (90 ± 9%) (Figure 6A). The exposure of PC12 cells to glutamate (20 mM) for 24 h also increased ROS production (166.7 ± 97.5%), which was prevented by concomitant treatment with EDAC (141.1 ± 38%) or coumarin (131.3 ± 49%) (Figure 6B).

## 4. Discussion

Previous studies have shown that extracts derived from *Amburana cearensis* exhibit important biological activities, including cytoprotective effects in models of neurodegenerative diseases [17,18,19,20,24] (Table 1). However, the bioactive compounds in EDAC with protective effects against oxygen and glucose deprivation-induced damage had not yet been identified [19]. The PC12 cell lineage has been a useful tool in studies of bioprospection of natural neuroprotective compounds against oxygen and glucose deprivation (OGD) [33,34]. In this study, we demonstrated that both EDAC and its isolated coumarin were able to protect PC12 cells subjected to 1 h of OGD followed by 24 h of reperfusion, whether treated concurrently with the compounds or receiving treatment after the damage. Thus, we highlighted the neuroprotective potential of EDAC and showed that coumarin is an important compound for triggering its protective action in models of oxygen and glucose deprivation. Glutamate-induced toxicity is a significant factor in cerebral ischemia [35]. Using the MTT cell viability assay, it was observed that 20 mM glutamate for 24 h reduced the viability in PC12 cell culture. Glutamate excess in the synaptic cleft can hyperactivate the NMDA and AMPA glutamate receptors on postsynaptic neurons, leading to calcium influx into these cells and subsequent activation of cell death [36]. Previous studies from our research group have demonstrated the neuroprotective effect of the dichloromethane extract of *Amburana cearensis* (EDAC) against glutamate excitotoxicity in various cell culture models, including PC12 cells [18], primary cerebellar cultures [24], and hippocampal slices [19]. However, the effect of isolated coumarin against glutamate excitotoxicity has not yet been investigated. In this work, we demonstrated that coumarin, the major compound in EDAC, mitigated the cell death induced by glutamate in PC12 cells, but it was less effective than EDAC, which totally inhibited glutamate cytotoxicity. Furthermore, we demonstrated that coumarin is an important compound for the neuroprotective effect of EDAC since it also protected PC12 cells against OGD and improved the same molecular mechanisms as induced by EDAC. Besides the main component coumarin, EDAC is composed of a minor proportion of methyl esters, γ-sitosterol and ethyl hexadecanoate [18]. Some neuroprotective effects have been demonstrated by these other compounds [37,38], which makes it impossible to confirm here that coumarin is the only neuroprotective compound in EDAC. Even if coumarin is the compound of major importance in the effects observed in the present study, further studies are required to clarify the possibility that the multiple compounds in EDAC have combinatorial or even synergistic effects.

To better characterize the protective effect of EDAC and coumarin, we analyzed the cleaved caspase-3 expression using immunofluorescence. Our results showed that EDAC and coumarin inhibit an increase in cleaved caspase-3 induced by glutamate in PC12 cells. When activated, caspase-3 initiates the caspase activation cascade, leading to cell death [39]. In studies with PC12 cells, it was shown that the inactivation of caspase 3 can occur through the suppression of the negative regulation of Bcl-2 and the positive regulation of Bax, as well as by preventing the release of mitochondrial cytochrome c into the cytoplasm, thereby inhibiting caspase activation [40]. An anticancer study analyzed the mechanism of coumarin-based derivatives showing that coumarin derivatives induce apoptosis by up-regulating caspase-3 and caspase-9 expression [41]. Osthol, a chemical compound derived from coumarin, was able to decrease Bax/Bcl-2 levels and increase PI3K/Akt-1 leading to cell survival against H_2_O_2_-induced cell damage [42].

A study demonstrated the protective effect of ethyl acetate extract (EAE) from *Arctium lappa* L. roots against glutamate-induced oxidative stress in PC12 cells by decreasing the expression of proteins such as Bcl-2/Bax and caspase-3. Assessment of antioxidant signaling pathways revealed reduced activation of p-p38, p-JNK, and p-ERK, indicating that the extract inhibited the phosphorylation of these proteins, thereby increasing cell viability [43]. Astragaloside IV (AGS-IV), the main compound in *Astragalus membranaceus*, was able to inhibit the increase of phosphorylated MAPKs Raf, MEK, and ERK levels in PC12 cells induced by glutamate by blocking their phosphorylation. This effect suggests that the MAPK pathway is an important regulator of neuronal apoptosis [44]. Our results support these findings by demonstrating that EDAC and isolated coumarin reduced the phosphorylation of ERK in PC12 cells. In addition to inhibiting phosphorylation, we also observed a decrease in total ERK1/2 levels, indicating that the compounds may also be acting to inhibit protein expression.

In vitro studies using PC12 cells have shown that antioxidants may reduce cellular damage in pathological conditions associated with excessive glutamate [45]. The antioxidant effect of natural compounds has been studied as a mechanism for neuroprotection in ischemia [43] and other neurological disorders [20]. Studies demonstrated the antioxidative effect of Amburoside A from trunk barks *A. cearensis* against 6-OGDA-induced oxidative stress [20], and the improvement of antioxidative compounds in the *A. cearensis* seeds induced by water restriction [23]. The antioxidative effect of coumarin from *A. cearensis* was previously evidenced in BV2 cells [17]. Coumarin, as well as amburoside A and vanillic acid obtained from a dry extract from a *A. cearensis* plant cultivated for seven months, demonstrated anti-inflammatory effect and free radical scavenging activity in BV2 cells [17]. The BV2 cell lineage is retroviral immortalized microglia derived from C57BL/6 mice [46]. Widely used in bioprospecting studies on neurological disorders since it expresses 90% positive for microglia cell markers containing macrophage-1 (MAC-1) and MAC-2 but negative for MAC-3 antigens [47]. BV-2 microglia can be stimulated in vitro to an inflammatory M1 phenotype under LPS treatment or to a regulatory M2 phenotype under IL-4/IL-13 treatment. This acknowledgment of in vitro polarization of BV2 cells has been useful in prospecting anti-inflammatory compounds [48]. On the other hand, the PC12 cell line was derived from a transplantable rat adrenal pheochromocytoma and was first described by Greene and Tischler in 1976 [49]. This cell line has similar characteristics to chromaffin cells, which have the capacity for moderate synthesis, storage, and secretion of catecholamines [49,50] and have been widely used as a tool for studying the function of neurons, neuronal differentiation, and neurotoxicity including induced by glutamate [51] and neuroprotection against glutamate excitotoxicity [52,53,54,55,56]. In this study, we observed that using DCFDA and CellROX assays for both EDAC and coumarin presents an antioxidant effect in PC12 cells against H_2_O_2_ or glutamate. The evidence of an antioxidative effect of coumarin in PC12 cells observed in this work highlighted the potential neuroprotective effect of this compound in the *A. cearensis* seeds and in EDAC. Our findings highlight coumarin as an important active component related to the neuroprotective action of EDAC, suggesting its potential for development as a therapeutic agent for ischemic and excitotoxic brain injuries.

## 5. Conclusions

This study investigated the in vitro effect of EDAC and coumarin and showed that coumarin is an important active component in *A. cearensis* seed for neuroprotection. Our findings provide evidence that both EDAC and coumarin present an in vitro neuroprotective effect, preventing damage in PC12 cells caused by OGD and glutamate excitotoxicity. EDAC and coumarin achieve that through the inhibition of caspase 3 activation, reduction of ERK1/2 and p-ERK expression, and antioxidant activity. These results encourage further preclinical studies to support a translational investigation.

## Figures and Tables

**Table 1:** Biological effects of *A. cearensis* compounds.

References	Compounds (Extracts or Isolated Compounds)	Experimental Models	Biological Activity
Pereira et al. [23]	- Cotyledons of Amburana cearensis - Phenolic compounds - Polyethylene glycol (PEG 8000) solution	- Seed imbibition conditions: Control: Water Water restriction: PEG 8000 solution at −1.2 and −1.4 MPa - Sampling time points: - 0, 24, 48, 72, and 96 h of imbibition - Experimental measurements: - Germinability evaluation - Radical scavenging ability - Total phenolic content analysis.	- Enhanced radical scavenging ability after 96 h of imbibition - Reduced water uptake - Decreased seed germinability - Faster biosynthesis of phenolic compounds under water restriction - Decreased total phenolic content compared to control - High correlation between total phenolics and antioxidant activity - Demonstrated adaptation mechanisms to harsh environmental conditions.
Lima Pereira et al. [18]	Seed extracts of Amburana cearensis: - Ethanolic extract - Hexane extract - Dichloromethane extract - Ethyl acetate extract - Predominant compound: Isoflavone coumarin Various compounds with antioxidant properties (identified by GC-MS)	- PC12 neural cell cultures - Cytotoxicity assessment: - MTT test Extract concentrations: 0.01–2000 μg/mL Exposure times: 24 and 72 h - Excitotoxicity model: Glutamate pre-treatment (1 mM for 6 h) - Extract concentrations: 0.1–1000 μg/mL Chemical profile analysis using GC-MS	- Hexane extract showed toxicity only at highest concentration (1000 μg/mL) after 72 h - All extracts increased cellular viability of PC12 cells - Protective effects against glutamate-induced neuronal damage - Neuroprotective potential attributed to antioxidant properties - No significant toxicity observed for most extracts - Increased resistance to glutamate-induced cell damage
Lima Pereira et al. [24]	Seed extracts of *Amburana cearensis*: - Ethanol extract (ETAC) - Hexane extract (EHAC) - Dichloromethane extract (EDAC) - Ethyl acetate extract (EAAC)	- Primary cerebellar cell cultures - In vitro model of excitotoxicity induced by glutamate (10 µM) - Isolated rat brain mitochondria - Oxidative stress model - Immunofluorescence analysis for: - Glutamine synthetase - β-III tubulin - GFAP (glial fibrillary acidic protein) - IBA1 (microglia marker)	- EHAC extract reduced cell viability by 30% after 72 h - Preservation of astrocytes, neurons, and microglia - ETAC extract protection of mitochondria: - Reduced mitochondrial swelling - Prevented membrane potential dissipation - Decreased reactive oxygen species (ROS) production - Reduced calcium influx - Neuroprotective potential against: - Oxidative stress - Glutamate-induced excitotoxicity - Potential therapeutic agent for neurodegenerative diseases
Oliveira et al. [25]	- Aqueous crude extract and fractions of Amburana cearensis seeds (CEFAC) - Phenolic compounds - Organic acids - Polyphenols (identified through LC-MS/MS-QTOF characterization)	- Microdilution method against strains of Candida albicans - Combination assay of CEFAC with Fluconazole - LC-MS/MS-QTOF for chemical characterization	- Antifungal activity with IC50 ranging from 45.6 to 2048 µg/mL against Candida albicans strains - Synergistic effect when combined with Fluconazole, resulting in decreased IC50 (1.81–11.9 µg/mL) - Potential antimicrobial modifying activity - Compounds in CEFAC demonstrated ability to interact and act synergistically with antimicrobial drugs
de Araújo et al. [17]	- Dry extract from Amburana cearensis (DEAC) - Coumarin (CM) - Vanillic acid (VA) - Amburoside A (AMB) - Chemical markers identified by HPLC-PDA: CM, AMB, and VA	- BV-2 microglial cell line - Lipopolysaccharide (LPS)-stimulated BV2 microglial cells. - MTT cytotoxicity test - Free radical scavenging activity assay - Nitric oxide (NO) production measurement (Griess reagent) - ELISA for cytokine levels - Western blot analysis for protein expression.	- No cytotoxicity observed at 5–100 μg/mL concentrations - Free radical scavenging activity against hydroxyl and superoxide radicals - Significant reduction of nitric oxide (NO) production - Decreased production of pro-inflammatory cytokines TNF-α and IL-6 - Reduced expression of inducible nitric oxide synthase (iNOS) - Suppression of MAPK signaling pathways (JNK and ERK1/2) - Anti-inflammatory effects in LPS-activated microglial cells - Potential neuroprotective activity - No significant effect on IL-10 levels - Did not suppress TLR-4 expression or NF-κB phosphorylation
Gouveia et al. [26]	- Ethanolic leaf extract of Amburana cearensis - Protocatechuic acid (detected by HPLC) - N-acetylcysteine (positive control)	- Mice model of cisplatin-induced ovarian toxicity - Analytical methods: High-performance liquid chromatography (HPLC) Histological analysis Immunohistochemistry Fluorescence analysis Protein immunoexpression evaluation	- Maintained percentage of normal follicles - Preserved cell proliferation - Reduced apoptosis - Decreased reactive oxygen species (ROS) concentrations - Increased glutathione (GSH) concentrations - Enhanced mitochondrial activity - Regulated p-PTEN and p-Akt protein expression - Prevented cisplatin-induced ovarian damage - Demonstrated antioxidant actions - Modulated phosphorylated PTEN and Akt protein expression
Ferreira et al. [19]	- Dichloromethane seed extract of Amburana cearensis (EDAC) rich in coumarin. - Chemical constituents characterized by 1H and 13C-NMR	Hippocampal brain slices from: - P6–8 and P90 Wistar rats - Transgenic mice (SOX10-EGFP and GFAP-EGFP) - Cell viability assay - Glutamate uptake test - Immunofluorescence analysis - Oxygen-glucose deprivation (OGD) model of ischemia.	- Increased expression of glutamate transporters - Enhanced glutamine synthetase expression - Protection against glutamate excitotoxicity - Prevented decrease in GFAP-EGFP in astrocytes - Increased astrocytic process branching - Protection of oligodendrocytes against process loss - Cytoprotective effects against ischemia - Modulation of astrocyte responses - Stimulation of glutamate homeostatic mechanisms
de Veras et al. [27]	- Essential oil extracted from the leaves of Amburana cearensis - Contains phytocomponents including germacrone.	- Acute toxicity test in mice - Formalin test for antinociceptive evaluation - Abdominal writhing induced by acetic acid - Carrageenan-induced peritonitis - Yeast-induced pyrexia - Carrageenan-induced paw inflammation - Histamine-induced paw inflammation.	- No acute toxicity observed at doses up to 2000 mg/kg; p.o. - Antinociceptive effect statistically equal to morphine - Analgesic activity in both neurogenic and inflammatory phases - Mechanisms of action: cholinergic system, adenosinergic system, and ATP-sensitive potassium channels (K-ATP) - Anti-inflammatory effects: reduction in TNF-α and IL-1β levels. - Reduced leukocyte migration in peritonitis model. - Antipyretic effect statistically superior to dipyrone. - Reduction in paw edema statistically superior to standard in both models (carrageenan and histamine).

## Data Availability

Data are contained within the article.
